# Effect of a Copaiba Oil-Based Dental Biomodifier on the Inhibition of Metalloproteinase in Adhesive Restoration

**DOI:** 10.1155/2021/8840570

**Published:** 2021-02-17

**Authors:** Eliane Avany Malveira Araújo, Geisy Rebouças Lima, Luciana Aleixo dos Santos de Melo, Leilane Bentes de Sousa, Marne Carvalho de Vasconcellos, Nikeila Chacon de Oliveira Conde, Carina Toda, Simone Assayag Hanan, Ary de Oliveira Alves Filho, Maria Fulgência Costa Lima Bandeira

**Affiliations:** ^1^School of Dentistry, Amazonas Federal University,UFAM, Manaus, AM, Brazil; ^2^School of Pharmaceutical Sciences, Amazonas Federal University,UFAM, Manaus, AM, Brazil

## Abstract

**Aim:**

This study sets out to evaluate the antiproteolytic activity of copaiba oil-based emulsion at the resin/dentin adhesive interface union formed with conventional and self-etching adhesives systems.

**Methods:**

At in situ zymography, 30 teeth were sectioned 2 mm below the enamel-dentin junction; a *smear layer* was standardized and subdivided into four groups. Gelatin conjugated with fluorescein was used and taken to the fluorescence microscope for evaluation. In cytotoxicity, the Trypan Blue method was used at four different time points. The tested groups were (G1) control with distilled water; (G2) 2% chlorhexidine (CLX); (G3) emulsion based on copaiba oil (EC) 10% + X; (G4) 10% EC + Y; and (G5) EC 10% alkaline. The zymographic assay used the same groups described, but in 30 seconds and 10 and 20 minutes. HT1080 cells were incubated and submitted to electrophoresis. The gel was analyzed using ImageJ software. Mann–Whitney and Kruskal–Wallis tests were used in the statistical analysis (*p* < 0.05).

**Results:**

ECs showed higher cell viability in the cytotoxicity test and showed a significant difference in 10 and 20 minutes. In the zymographic assay, alkaline EC reduced 67% of MMP-2 activity and 44% of MMP-9 compared to 2% chlorhexidine. At in situ zymography in qualitative evaluation, all groups tested showed inhibition of activity in metalloproteinases.

**Conclusion:**

EC showed activity in the inhibition of metalloproteinases *in vitro* and in situ, especially the alkaline one. The survey shows the possibility of using ECs, a product from Amazonian biodiversity, as a biomodifier in dentistry.

## 1. Introduction

During the restorative treatment, the adhesion of the material to the dentin substrate occurs when the adhesive infiltrates the demineralized dentin layer by acid conditioning, encapsulating the exposed collagen and protecting the adhesive interface from proteolytic and hydrolytic degradation. This layer can change its longevity because that collagen, present in the hybrid layer, is susceptible to hydrolysis and proteolytic action [1–3]. Metalloproteinases are activated calcium-dependent and zinc enzymes and are naturally trapped in mineralized dentin during odontogenesis. However, they can be activated during previous acid conditioning or in acidic monomers present in the self-etching system [4–8].

There are several strategies to stabilize this interface, such as nonselective inhibitors, whose example is Chlorhexidine; physical agents such as UV light; unspecific synthetic collagen crosslinking agents such as glutaraldehyde or cytotoxic carbodiimide (EDC); and biomodifiers such as proanthocyanidin extracts obtained from grape seed, cocoa seed, cranberry, cinnamon, and açaí [5, 9–11]. Copaiba, whose oil has been the subject of research, shows a promising solution, exposing significant results in cavity cleaning, bactericidal, and bacteriostatic potential similar to the 2% chlorhexidine digluconate solution [12]. Studies have shown its efficiency in maintaining adhesive strength [13], presenting a homogeneous, regular, continuous hybrid layer, and the presence of tags independent of the dentin substrate [13, 14].

Therefore, this study aimed to evaluate the antiproteolytic activity of copaiba oil-based emulsion compared to CLX solution at the union of the resin/dentin adhesive interface formed with the conventional and self-etching adhesive system.

## 2. Methodology

The project was approved by the Human Research Ethics Committee (CEP) of the Federal University of Amazonas (UFAM), with CAAE number 35573914.0.0000.5020. The oil-resin from *Copaifera multijuga* was extracted from the National Institute of Amazon Research (INPA, Amazônia, Brazil), and the exsiccate was deposited in the INPA herbarium under No. 270709. The copaiba emulsions were prepared by varying only the concentration of the preservative diazolidinyl urea: EC + X; EC + Y; and EC alkaline [12].

### 2.1. Cytotoxicity Test

The cytotoxicity of the substances was tested against the HT1080 strain using the Trypan Blue test in quadruplicate. The HT1080 strain was grown in Dulbecco's Modified Eagle Medium (DMEM, Ref. 12100046, Invitrogen), supplemented with 10% Fetal Bovine Serum (FBS, Gibco) and kept in an oven at 37°C and an atmosphere containing 5% CO_2_. The cells (2 × 105 cells/well) were plated in a 24-well plate to receive the treatments CLX, EC + X, EC + Y, alkaline EC, and Phosphate-Buffered Saline (PBS) as the negative control, in the periods of 15 seconds, 30 seconds, 10 minutes, and 20 minutes. Then, the samples were removed from the plate, divided into microtubes, and centrifuged for 4 minutes.

After centrifuging, part of the supernatant was dispensed, the cells were resuspended, and 10 *μ*L of stock solution of Trypan Blue (T6146, Sigma-Aldrich, 0.4% in PBS) was added. The dye remained in contact with the cells for about 1 minute, and then 10 *μ*L was transferred to the Neubauer Counting Chamber (Marienfeld) for counting cells under a microscope.

### 2.2. Cell Zymography

The zymographic method was used to evaluate the activity of matrix metalloproteinases (MMP-9 and MMP-2) present in the HT1080 cell line [15]. For the assay, HT1080 cells were transferred to 24-well plates at a concentration of 20 × 10^4^ cells/well and incubated in an oven. After 24 hours, the cells were treated according to the groups: G1: distilled water; G2: 2% chlorhexidine digluconate; G3: emulsion based on 10% copaiba oil (EC) + Preservative X; G4: EC at 10% + Preservative Y; G5: EC at 10% alkaline and PBS (positive control), in 30 seconds, 10 minutes, and 20 minutes. After each treatment time, the culture medium was removed and centrifuged at 5000 rpm/10 minutes. For protein separation, sodium dodecyl sulfate and polyacrylamide gel (SDS- PAGE gel) with 10% acrylamide and 1% gelatin were used, in which 10 *μ*g of protein per channel was added. The gel was subjected to electrophoresis (120 volts for 1 hour, 30 minutes) for protein separation. After that, the gel was washed twice for 20 minutes, with 2.5% Triton X-100. Then, the gel was incubated with incubation buffer (0.05 M TrisHCl pH 8, 5 mM CaCl_2_, 5 *μ*M ZnCl_2_), overnight, at 37°C, for substrate degradation by MMPs. Soon after, the gel was stained with Coomassie Brilliant Blue G-250 (0.5% Coomassie blue in 30% methanol and 10% acetic acid) for 30 minutes, at room temperature, under constant agitation, and bleached with 10% methanol and 10% acetic acid. The images of the bands were compared and analyzed using the Image J software. After data collection, a descriptive analysis of the results was performed.

### 2.3. In Situ Zymography

Thirty fully included and newly extracted human third molars were used; these teeth were extracted for orthodontic, prosthetic, or surgical reasons. They were frozen immediately and stored until the moment of use [16].

All teeth were sectioned 2 mm below the central region of the occlusal surface to remove the enamel. The root with the aid of a 0.5 mm thick diamond wheel was adapted on the Mecatome® P100 cutting machine, under refrigeration. The cut occlusal portion was not used in the research [17]. Then, the dentin surfaces were abraded with the aid of 320 grit silicon carbide sandpapers, under constant cooling, at a speed of 600 rpm. They were adapted to an AROTEC® polisher to expose a flat dentin surface [4].

The surface treatment of all groups was standardized, changing only the agent used [13]. The following agents were used according to the groups described; afterward, the teeth were restored 1 mm thick with Filtek Z350 XT® resin; then, the specimens were cut vertically to 1 mm thick to expose adhesive/dentin and placed on a blade; after that, further cuts were made until 50 *μ*m of thickness was obtained. In this test, the emulsion was chosen with the best results in the cytotoxicity test and the zymography in cells, so the alkaline EC was used, which has a pH of 8.3. Group 1 = alkaline EC treatment + conventional adhesive system (Adper™ Single Bond 2); Group 2 = alkaline EC treatment + self-etching adhesive system (Clearfil™ SE Bond); Group 3 = 2% chlorhexidine digluconate + conventional adhesive system (Adper™ Single Bond 2); Group 4 = 2% chlorhexidine digluconate + self-etching adhesive system (Clearfil™ SE Bond).

In situ zymography was performed with gelatin conjugated to fluorescein as a MMP substrate (E-12055, Molecular Probes®, Eugene, OR, USA) [16]. Afterward, the gelatin was poured over each slide and covered with a coverslip and taken to humidified chambers at 37°C for 24 hours protected against light. Hydrolysis of the gelatin substrate conjugated to extinct fluorescein indicated endogenous gelatinolytic enzyme activity and was evaluated by a fluorescence microscope. The negative control of enzymatic activity was incubated in the same way, using ethylenediaminetetraacetic acid (EDTA) on the other half of the tooth. It is a well-known MMP inhibitor. EDTA is a chelator, widely used as a negative control in zymographic tests for its effectiveness in inhibiting MMPs [16].

### 2.4. Statistical Analysis

In the cytotoxicity test, data were presented using graphs and tables, where the median and quartiles (Qi) were calculated, as the hypothesis of normality of the data was rejected using of the Shapiro–Wilk test. In the comparison of medians, the Kruskal–Wallis non-parametric test was applied to compare more than two groups and Mann–Whitney for only two groups [18]. In the zymography test in cells, the bands' images were compared and analyzed using the ImageJ software.

In the in situ, zymography test, data were presented using tables, where medians and interquartile difference (di) were calculated since the hypothesis of normality using the Shapiro–Wilk test was rejected. When comparing the medians, the Mann–Whitney non-parametric test was applied for two medians and Kruskal–Wallis for more than two medians [18]. The software used in the data analysis was the program Minitab® version 18 for Windows®, and the level of significance set in the statistical tests was 5%.

## 3. Results of Cytotoxicity Test

**Figure 1:**
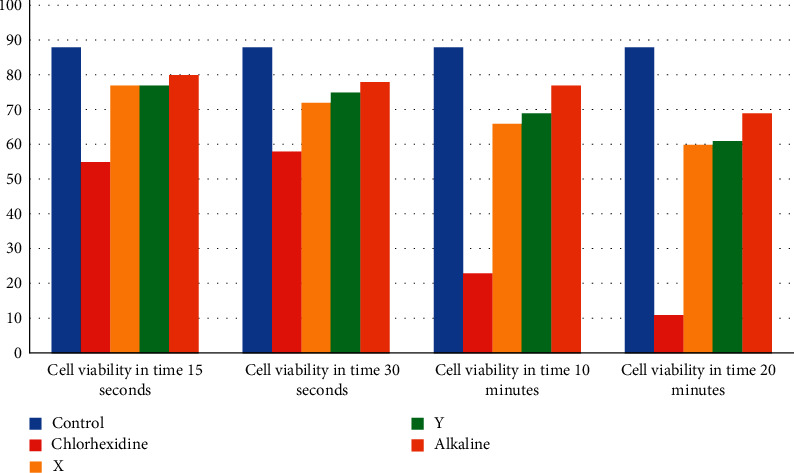
Percentage of the tested substances cell viability within 15 seconds and 30 seconds; there was no significant difference. However, it is observed that the lowest cell viability was of CLX at 2%; 10 and 20 minutes, with the test emulsions being similar to the control group, end the CLX at 2% different from the others.

In the times of 15 seconds, 30 seconds, 10 minutes, and 20 minutes, the percentage of cell viability was obtained in the cytotoxicity assay results performed by the Trypan Blue test (Figure 1).

Table 1 shows that, at 15 seconds and 30 seconds, there was no significant difference between the experimental groups (*p*=0.055 and 0.424, respectively). However, it is observed that the lowest cell viability was of CLX at 2%. At 10 and 20 minutes, there was a significant difference in the groups as a function of time, with the test emulsions being similar to the control group, and the CLX at 2% different from the others.

### 3.1. Cell Zymography

**Figure 2:**
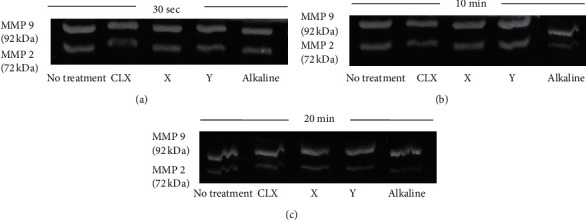
Bands of enzymatic degradation by MMP-2 and MMP-9, in all groups tested, in the periods of 30 seconds (a), 10 minutes (b), and 20 minutes (c).

In the zymographic analysis, after staining, it was possible to visualize the bands of enzymatic degradation in the gels (Figure 2).

**Figure 3:**
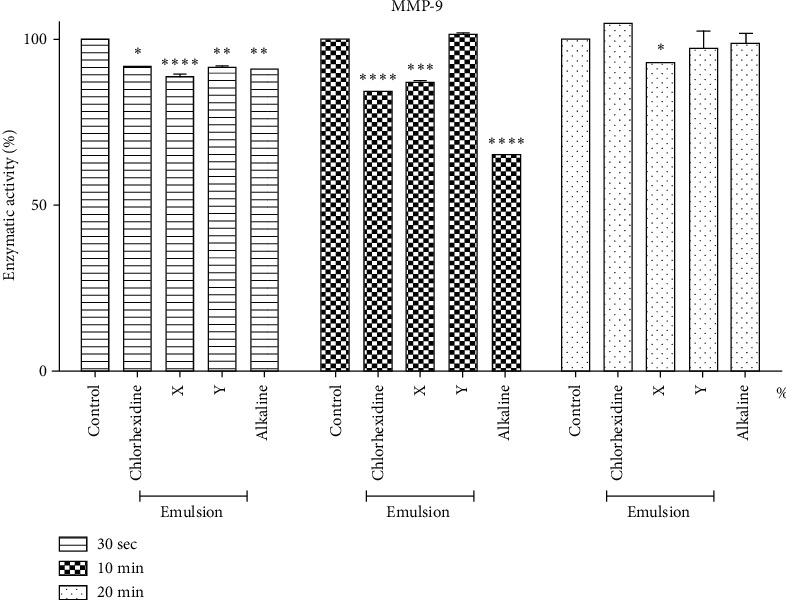
Analysis of the enzyme activity of MMP-9 compared to treatments with a tested solution. It shows, over 30 seconds, a statistical difference in the decrease in MMP-9 activity among all tested solutions when compared to the control group without treatment. In 10 minutes, there was a statistical difference only between CLX, EC + X, and alkaline EC. At 20 minutes, the only solution that showed a significant difference in decreasing MMP-9 activity was EC + X.

Figure 3 shows a statistical difference in the decrease in MMP-9 activity among all tested solutions compared to the control group without treatment, over 30 seconds. In 10 minutes, there was a statistical difference only between CLX, EC + X, and alkaline EC. At 20 minutes, the only solution that showed a significant difference in decreasing MMP-9 activity was EC + X. Analyzing the data processed by the ImageJ software, the best result was alkaline EC in 10 minutes, about 35% decreased activity of MMP-9.

**Figure 4:**
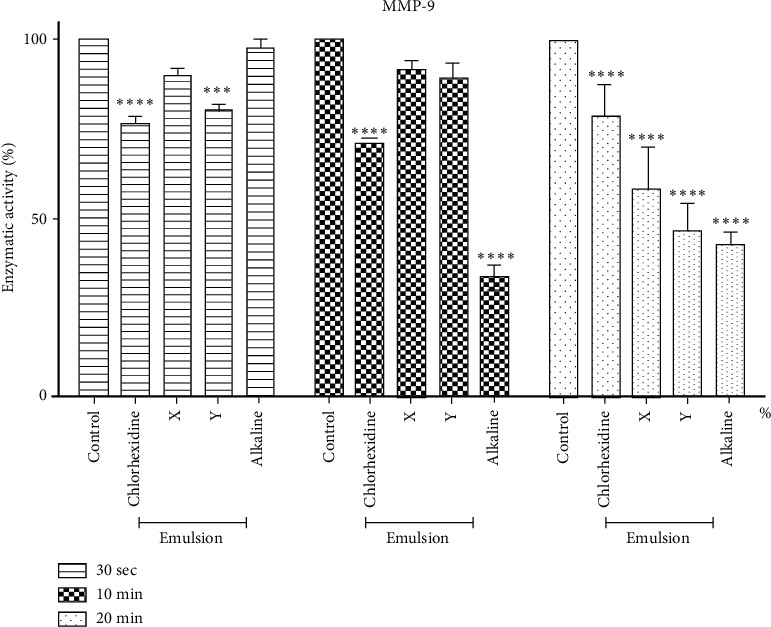
Analysis of the enzyme activity of MMP-2 compared to treatments with tested solutions. It shows the decrease in enzyme activity in MMP-2. In the time of 30 seconds, there was a statistical difference between the CLX solution and the EC + X when compared to the positive control. Within 10 minutes, a statistical difference was observed between CLX solution and alkaline EC, each showing about 44% and 67% decrease in enzyme activity, respectively. Finally, at 20 minutes, there was a statistical difference between CLX solution, EC + X EC + Y, and 10% alkaline EC.

Figure 4 shows the decrease in enzyme activity in MMP-2. In 30 seconds, there was a statistical difference between the CLX solution and the EC + X, compared to the positive control. Within 10 minutes, a statistical difference was observed between CLX solution and alkaline EC, each showing about 44% and 67% decrease in enzyme activity, respectively. Finally, at 20 minutes, there was a statistical difference between CLX solution, EC + X, EC + Y, and 10% alkaline EC.

Carrying out a comparative analysis of the biological activities of ECs, it was observed that the 10% alkaline emulsion showed better biological responses and decreased enzyme activity of MMP-2 and -9.

### 3.2. In Situ Zymography

**Figure 5:**
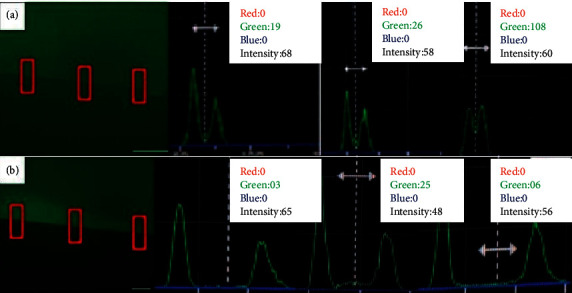
Histograms of MMP activity: (a) chlorhexidine digluconate with Clearfil™ and (b) chlorhexidine with Adper Single Bond 2™.

**Figure 6:**
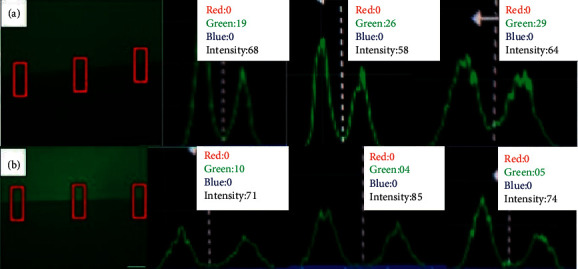
Histograms of MMP activity: (a) alkaline emulsion with Clearfil™ and (b) alkaline emulsion with Adper Single Bond 2™.

**Figure 7:**
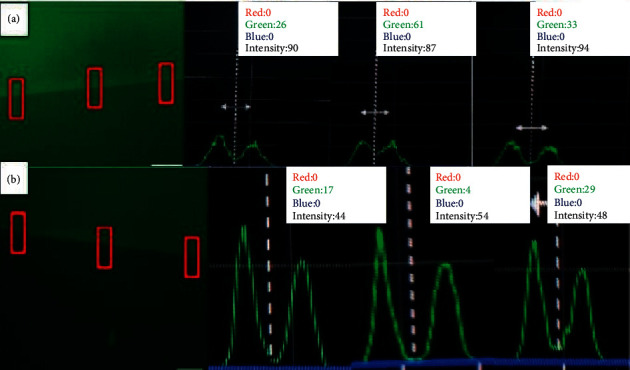
Histograms of MMP activity: (a) EDTA with Clearfil™ and (b) EDTA with Adper Single Bond 2™.

The analysis under the fluorescence microscope showed inhibition of MMP caused by the gelatin's degradation, and the fluorophore that was bound to the gelatin is released and emits fluorescence, a fact evidenced in the histograms in Figures 5–7. The statistical analysis is represented in Tables 2–4.

Table 2 demonstrates that none of the experimental groups showed a statistical difference using the Clearfil™ adhesive system.

Table 3 shows that, in the Adper™ Single Bond adhesive system 2, there was a statistical difference in the emulsion concerning the control group, and it was similar to CLX.

Table 4 shows that when comparing adhesive systems and test products, there was no statistical difference between the experimental groups, except in the control group.

## 4. Discussion

MMPs are calcium- and zinc-dependent endopeptidases whose function is to degrade the extracellular matrix, tissue remodeling, and angiogenesis. They are secreted in the form of zymogens [19]. MMP-2 and MMP-9 are the most prevalent in dentin, the first being in greater quantity [6–8].

The failures in the resin/dentin layer are related to the degradation that the MMPs promote. During the procedure of adhesion of the restorative material to the dental structure, collagens are found exposed in a region with the presence of water where the adhesive cannot encapsulate this collagen due to its hydrophobic nature [2, 4–6, 8, 10, 20, 21].

CLX 2% is efficient, in the short term, in maintaining the dentin-resin bond strength, improving the quality in the hybrid layer longevity; due to the action on MMPs and cathepsins-cysteines, the union of CLX with dentin is of the reversible electrostatic type. Its activity depends on CLX's substantivity of; on average, this lasts for 180 months [1, 8, 22–24]; however, its cytotoxic potential is known, so it is not advisable to use it in deep layers, close to the dental pulp.

The cytotoxicity test is performed to clarify whether the test product is cytotoxic or not to a specific cell. Initially, in this study, this test was carried out to verify the viability of the materials against HT1080 cells, and subsequently to perform the zymographic analysis to assess the antiproteolytic activity.

In the tested times, the ECs showed a higher percentage of cell viability when compared to CLX and similar to the control. However, a statistical difference was found with CLX only in the 10 and 20 minutes times [25, 26]. Conflicting results were observed in a study with copaiba oil *in nature,* in which only 30% of the cells remained viable when compared to the control group [27]. In addition to the cytotoxicity test, the ECs do not reach the cells' DNA in tested concentrations, showing that this emulsion is neither cytotoxic nor genotoxic [26].

About the enzymatic activity, in the time of 30 seconds, the MMP-2 was inhibited by the solutions of CLX and EC + X, with a statistical difference when compared between themselves and the control group. Within 10 minutes, a statistical difference was observed between CLX solution and EC at 10% alkaline, each showing about 44% and 67% decreased enzyme activity, respectively. In 20 minutes, there was a statistical difference between CLX solution, EC + X, and EC + Y, with EC being alkaline with higher inactivity in MMPs.

Within 30 seconds, all tested solutions showed a decrease in MMP-9 activity, with a statistical difference compared to the control group. In 10 minutes, there was a statistical difference only between CLX, EC + X, and alkaline EC. At 20 minutes, the only solution that showed a significant difference in decreasing MMP-9 activity was EC + X. Analyzing the data, the best result was alkaline EC in 10 minutes, with a 35% decrease in the antiproteolytic action of MMP-9.

We observed that CLX decreases MMPs' activity in cell culture zymography assay, sequestering calcium, and zinc ions making it challenging to activate endopeptidases [1, 22, 28–30]. Based on the results of this study, it can be suggested that the tested emulsions also decrease the proteolytic activity of MMPs.

Since this is an original work about the use of the emulsion as a biomodifier, there are no reports in the literature that can support the findings of the present study.

In this sense, some studies used copaiba-based emulsions as a biomodifier with a potential similar to that of CLX [13, 31]. These emulsions also showed satisfactory antimicrobial activity against bacterium found in the oral cavity [25], as a bioactive for cleaning cavities [12].

Besides, it was observed through the histopathological study and electron microscopy a homogeneous and continuous hybrid layer with resin tags in healthy and affected by caries dentine with EC treatment before to conventional and self-etching adhesive systems use [31], as well as an improvement in adhesive strength [13, 32, 33].

ECs could act as a natural crosslinking, just like proanthocyanidin (PA) compounds. PA belonged to a condensed tannins group and can be found in fruits, vegetables, peels, etc. The grape seed extract, cocoa, cranberry, cinnamon, and açaí were studied and presented good tensile strength, modulus of elasticity, and resistance to biodegradation and to demineralization [8, 10, 21].

Several products have been produced to minimize the action of MMP and act as biomodifiers or as collagen crosslinking agents [8, 21, 34, 35], such as green tea extract, Galardina,1-ethyl- 3carbodiimide (EDC), grape seed extract, and quercetin [5, 9–11].

In the microscopic analysis of the present study, no intense fluorescence was noticed in the groups tested, with results similar to the EDTA group, however, only in the Adper Single Bond 2 adhesive system was there a statistical difference in the emulsion concerning to the control group, being similar to chlorhexidine digluconate 2%. EDTA causes metal ions' chelation, removing calcium from the dental structure, being one of the negative controls most used in studies of MMPs [5, 6, 8, 19].

The activity increasing of these enzymes occurs both in conventional and in self-etchers adhesive systems, although in a smaller proportion in the self-etcher, due to the characteristic of the hybrid layer formed (less thick) [2, 4–6, 10, 20]. In this study, only the Adper Single Bond 2 adhesive system group shows a statistical difference. All the studies presented in the literature are qualitative analysis, as they only assess whether MMPs are inhibited or not. This research quantified the antiproteolytic activity using software, allowing statistical analysis.

The alkaline emulsion (pH 8.3), tested in situ, showed the best result in the viability and zymographic tests once MMPs usually are activated at a low pH due to adhesive systems, acid conditioning, or even the carious process [5, 6, 8].

There are conflicts in the literature about the effect of pH on the MMPs. The phosphoric acid chemically denatures these enzymes [28]. However, the acid etching in dentin promotes a reaction between acid phosphate ions and calcium, forming a calcium phosphate precipitate (CaHPO_4_), which temporarily hides MMPs action [36]. Still, below this layer, MMPs are activated [19].

There are three possibilities to the in vitro and in situ antiproteolytic activity of ECs on MMPs: the high molecular weight of copaiba oil can decrease the spaces formed between the collagen fibers, once the adhesives solvents, especially ethanol, are unable to occupy this region [8]; the form of its presentation, because the oil can waterproof the collagen fibers, favoring the penetration of hydrophobic adhesive system between the fibers protecting them from the MMPs action and forming a waterproof hybrid layer; and finally, the effect of pH, as reported above.

The results of this study indicated the possibility of using copaiba oil emulsions as a biomodifier; however, other in situ and *in vivo* studies are needed to demonstrate the clinical emulsions' efficacy.

## 5. Conclusion

The emulsions were similar to the control group in the cytotoxicity test at different preservative levels and treatment times. All concentrations of emulsions showed activity on MMP-2 and MMP-9, but the one that showed the most significant inhibition was alkaline EC in 10 min. The ECs showed inhibition activity of metalloproteinases 2 and 9 at the resin/tooth adhesive interface in the in situ study with cell viability expressed by cells of the HT1080 lineage, highlighting the alkaline EC. This study, combined with previous research, provides further evidence of the possibility, in the future, of using a product from Amazonian biodiversity in dentistry, requiring clinical and long-term studies to achieve the effectiveness of copaiba oil emulsions in adhesive restorations.

## Figures and Tables

**Table 1:** Comparison of medians of viable cells in relation to groups according to time.

Groups	Time											
	15 s			30 s			10 m			20 m		
	Q1	Med	Q3	Q1	Med	Q3	Q1	Med	Q3	Q1	Med	Q3
X	69.5	103.5	133.0	71.0	92.0	111.5	54.5	107.0^a^	153.5	30.5	55.5^a^	86.5
Y	57.5	66.5	89.0	58.0	83.0	121.5	53.5	73.0^a^	96.5	24.5	35.5^a^	51.5
Alkaline	59.5	76.0	83.5	49.5	66.5	82.5	50.5	86.5^a^	122.5	44.5	62.5^a^	81.0
Chlorhexidine	24.5	31.0	44.0	35.5	57.0	85.0	6.5	17.5^b^	28.5	0.5	3.0^b^	8.0
Control	58.0	97.0	139.0	58.0	97.0	139.0	58.0	97.0^a^	139.0	58.0	97.0^a^	139.0
*p*	550			424			41			13		

Med: median; Qi: quartiles. Different letters indicate statistical difference at the 5% level of significance.

**Table 2:** Comparison in relation to the experimental groups for each measure of the Clearfil™ adhesive system, Manaus, AM.

Groups	Measure					
	Left		Middle		Right	
	Med	Di	Med	Di	Med	Di
Negative control	57.0	62.0	46.0	41.8	76.0	100.0
Chlorhexidine	37.0	58.5	25.0	19.5	113.0	101.0
Emulsion	37.5	73.5	33.5	79.3	98.5	74.5
*p* ^*∗*^	0.668		0.467		0.874	

Med: median; Di: interquartile deviation; ^*∗*^Kruskal–Wallis test.

**Table 3:** Comparison in relation to groups for each measure of the Adper Single Bond 2™ adhesive system, Manaus, AM.

Groups	Measure					
	Left		Middle		Right	
	Med	Di	Med	Di	Med	Di
Negative control	4.5	30.0	5.5	26.2	11.5^a^	19.2
Chlorhexidine	4.0	39.8	12.0	18.0	23.5^ab^	47.3
Emulsion	53.5	89.3	50.5	99.8	56.5^b^	75.0
*p* ^*∗*^	0.124		0.272		0.040	

Med: median; Di: interquartile deviation; ^*∗*^Kruskal–Wallis test.

**Table 4:** Comparison in relation to adhesive systems according to measures and groups, Manaus, AM.

Measures/groups	Adhesives				
	Clearfil⟶		single bond 2	
	Med	di	Med	di	*p* ^*∗*^
*Left*					
Negative control	57.0	62.0	4.5	30.0	83
Chlorhexidine	37.0	58.5	4.0	39.8	81
Emulsion	37.5	73.5	53.5	89.3	773
					
*Middle*					
Negative control	46.0	41.8	5.5	26.2	81
Chlorhexidine	25.0	19.5	12.0	18.0	78
Emulsion	33.5	79.3	50.5	99.8	884
					
*Right*					
Negative control	76.0	100.0	11.5	19.2	*43*
Chlorhexidine	113.0	101.0	23.5	47.3	149
Emulsion	98.5	74.5	56.5	75.0	773

Med: median; Di: interquartile deviation; ^*∗*^Kruskal–Wallis test.

## Data Availability

The concentration of the mixtures data used to support the findings of this study have not been made available because of legal concerns, such as commercial confidentiality.
